# From neuroinflammation to neural inference: computational psychiatry meets precision medicine

**DOI:** 10.1038/s41537-026-00757-8

**Published:** 2026-04-22

**Authors:** Ghaith K. Mansour, Ahmad W. Hajjar

**Affiliations:** 1 College of Pharmacy, Alfaisal University, Riyadh, Saudi Arabia; 2 College of Medicine, Alfaisal University, Riyadh, Saudi Arabia

**Keywords:** Biomarkers, Schizophrenia

## Abstract

Psychosis spectrum disorders exhibit substantial clinical variability, necessitating novel frameworks that transcend traditional symptomatic classification. While polygenic risk accounts for significant heritability, the precise mechanisms translating genetic vulnerability into clinical illness remain elusive. An increasingly compelling hypothesis suggests that immune-related abnormalities link genetic risk with environmental stressors to precipitate neural dysfunction. This review integrates neuroimmunology and computational psychiatry to advance mechanism-driven precision medicine by connecting specific molecular dysfunctions to high-level information processing deficits. We synthesize evidence demonstrating that genetic and environmental risks converge on immune pathways—particularly microglial dysfunction and aberrant synaptic pruning. Functionally, we propose this pathology drives fundamental information processing errors, including maladaptive prior beliefs and reduced sensory precision. Here we highlight the necessity of multi-modal biomarkers and real-time digital phenotyping to stratify patients based on underlying neuro-immune endotypes. Finally, we address the critical challenge of algorithmic bias, emphasizing that proactive, standards-based strategies are required to ensure computational models are equitable and generalizable across diverse global populations. This integrated roadmap offers a path toward a truly personalized, biologically grounded psychiatry.

## Redefining Psychosis through Neuro-Immune and Computational Lenses

The global burden of mental illness is increasingly dominated by the staggering heterogeneity of the psychosis spectrum^1,2^, a domain where traditional symptom-based diagnoses often fail to capture the underlying biological reality^3^. For decades, clinical practice has relied on the cross-sectional observation of phenomenological traits—such as hallucinations, delusions, and disorganized thought—to categorize complex neurodevelopmental trajectories^4,5^. However, as the field moves into the era of precision medicine^6^. This shift requires a departure from static diagnostic categories toward a dynamic understanding of how biological dysregulation manifests as clinical illness^7,8^. This clinical landscape is further complicated by the fact that individuals sharing a single diagnosis, such as schizophrenia, often exhibit vastly different neuroanatomical and genetic profiles. This “diagnostic silence” regarding underlying biology has historically hindered the development of targeted therapies, as treatments are frequently applied based on broad symptom clusters rather than being matched to the specific biotype of the individual patient^9^.

Central to this challenge is the intricate interplay between the immune system and neural circuitry—a relationship that determines the very architecture of human perception and information processing^10^. While polygenic risk accounts for a substantial portion of the heritability observed in these conditions, reaching up to 80% for schizophrenia^11,12^ the exact biological mechanisms that translate this genetic vulnerability into clinical illness remain mysterious^13^. Genetic risk alone is rarely deterministic; instead, it creates a landscape of vulnerability that interacts with environmental stressors throughout development. New conceptual frameworks are urgently required to connect these diverse gene-environment interactions to the underlying pathophysiological processes that disrupt neural stability^14^. An increasingly compelling hypothesis suggests that immune-related abnormalities serve as the critical biological pathways that functionally link high polygenic genetic vulnerability with environmental stressors to precipitate neural dysfunction^15,16^.

A significant portion of the polygenic risk for schizophrenia originates in neuronal-specific pathways, such as those governing synaptic plasticity^17^ and calcium signaling^18,19^. Therefore, microglial dysregulation should be viewed as a critical secondary pathway or a ‘transducer’ of risk in a specific subset of patients, rather than a universal mechanism for the entire psychosis spectrum^20^. This necessitates ongoing efforts to stratify cases based on both peripheral and cerebrospinal fluid (CSF) inflammatory markers to identify those for whom neuro-immune mechanisms are the primary driver of illness^21,22^. In this view, the immune system acts as a transducer, converting external environmental “hits”—such as prenatal infection or adolescent stress—into cellular signals that reshape the brain’s architecture. These environmental “hits” trigger a cascade of molecular events that can “prime” the brain’s resident immune cells. This priming makes the system hyper-responsive to later challenges, potentially accelerating the transition from a state of vulnerability to clinical psychosis during critical neurodevelopmental windows^23^.

Concurrently, the field of computational psychiatry has emerged as a rigorous, quantitative language to model how this biological dysregulation translates into measurable errors in information processing. Rather than viewing symptoms as isolated occurrences, this framework offers an information processing-based account of psychosis^24^, suggesting that alterations in exteroception (self-environment interaction) and interoception (self-self interaction) lead to the emergence of positive symptoms^25^. In this context, interoception refers to the brain’s internal monitoring of its own signals, such as the ability to predict the sensory consequences of one’s own thoughts^26,27^; a failure here leads to a lost sense of agency (e.g., thought insertion)^28,29^. Conversely, exteroception refers to how the brain integrates external sensory data into its internal world model. When this interaction is disrupted, the brain fails to update its beliefs based on reality, leading to the formation of delusions^30,31^. This computational lens treats the brain as an inference engine that constantly generates predictions about the world^32,33^.

The integration of these two powerful domains—neuroimmunology and computational science—is a necessary step toward mechanism-driven, precision psychiatry^34,35^. To achieve this, the field has adopted sophisticated methods for systematic investigation, such as those promoted by the Bipolar Schizophrenia Network on Intermediate Phenotypes (BSNIP) project. This multi-level approach is essential for identifying the precise “intermediate phenotypes” that bridge the gap between a risk gene and a clinical symptom^36,37^. To generate actionable clinical pathways, we must specifically define the computational consequences of synaptic loss^38^. One of the most promising concepts in this area is precision weighting, which refers to the mathematical “confidence” or gain assigned to sensory information versus internal prior beliefs^39^. In a healthy brain, excitatory synapses provide the high-fidelity signaling necessary to communicate “prediction errors”—the difference between what we expect and what we perceive^40,41^. We propose that microglial-mediated pruning of excitatory synapses in the prefrontal cortex (PFC) reduces the synaptic gain required to signal new sensory data, leading to reduced sensory precision^42–44^. This creates a computational imbalance where internal models are never corrected by external reality, essentially trapping the individual in a self-generated perceptual loop that manifests as clinical delusions^45^. Consequently, the brain is forced to over-rely on “maladaptive priors” to explain the environment. This mechanism moves the field beyond descriptive symptoms toward quantifying fundamental alterations in neural circuitry underlying Cognitive Systems^45^ (Table 1).

**Table 1:** Mechanistic Integration of Neuro-Immune Activity and Computational Deficits.

Neurobiological Mechanism	Biological Finding	Predictive Coding Concept	Computational/Clinical Manifestation
Aberrant Synaptic Pruning	C4 variants drive excessive microglial elimination of excitatory synapses in the PFC.	Reduced Sensory Precision	Reduced synaptic gain required to signal new sensory data.
Circuit Imbalance	Specific loss of excitatory inputs disrupts the signal-to-noise ratio in cortical microcircuits.	Prediction Error Failure	The brain fails to distinguish meaningful environmental cues from internal neural fluctuations.
Circadian/Sleep Disruption	Sleep disturbances alter microglial states and trigger peripheral immune shifts.	Inference Instability	A marked decrease in environmental predictability prevents accurate weighing of sensory precision in real-time.

**Fig. 1:**
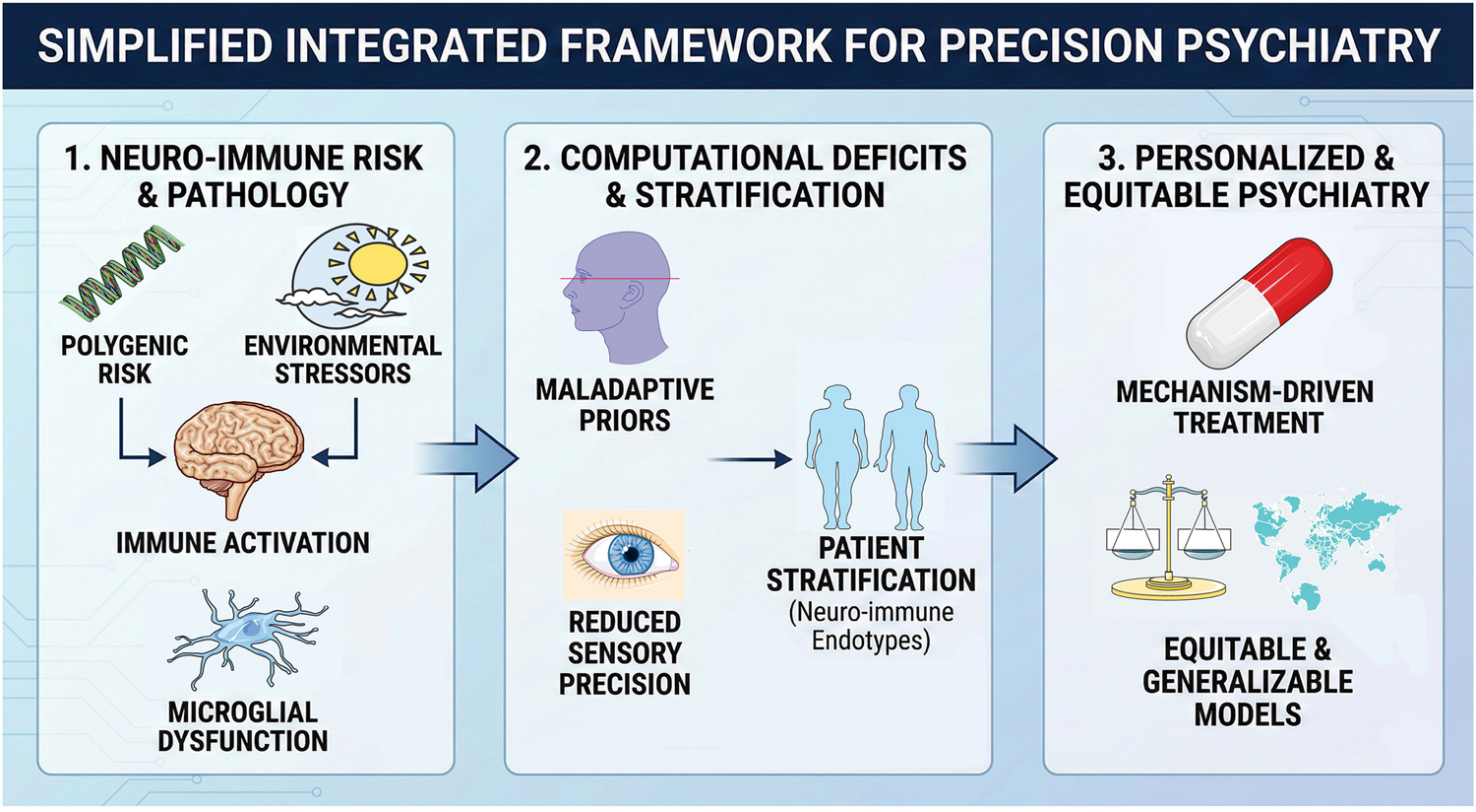
Simplified Integrated Framework for Precision Psychiatry. This roadmap illustrates the convergence of genetic vulnerability and environmental stressors on immune pathways, particularly microglial dysfunction (1). This pathology drives computational errors, such as reduced sensory precision and maladaptive priors, which serve as endotypes for patient stratification (2). These insights enable a transition toward mechanism-driven treatments and the implementation of equitable, generalizable predictive models (3).

While we know that immune markers are elevated^46^ and that computational parameters are shifted^47^, a critical research gap remains: we lack a unified framework that explains how cellular-level immune activity, such as microglial-mediated pruning, manifests as the specific computational errors that define the psychotic experience^48^. Bridging this gap requires moving beyond static, “one-size-fits-all” neurobiological models^49^. We must instead adopt dynamic, multi-modal approaches that can capture the non-linear interactions between immune-active states and the resulting shifts in cognitive processing^50^. This review addresses this gap by synthesizing recent evidence from neuroimmunology, machine learning, and digital health. Our objective is to demonstrate how biological deficits can be operationalized through computational models and real-time digital biomarkers to create a personalized roadmap for care (Fig. 1). In the following sections, we detail the molecular mechanisms of microglial dysregulation before demonstrating how these deficits disrupt cortical circuitry, leading to the computational failures observable in clinical populations.

## Molecular Mechanisms: Microglial Dysregulation and Synaptic Pathology

While the extended Major Histocompatibility Complex (MHC) represents a robust individual genetic signal in schizophrenia^51,52^. However, the immune system remains a significant secondary pathway, where polygenic risk likely modulates how microglia respond to neurodevelopmental stressors^20^. A finding with substantial functional implication involves the complement component 4 (C4) gene, where distinct, common functional forms contribute significantly to the overall risk of schizophrenia^51^. Evidence for immune system involvement spans the periphery^53^ (and the central nervous system (CNS)^54^. Peripheral analysis consistently reveals elevated levels of proinflammatory cytokines, including Interleukin-1β (IL-1β), Interleukin-6 (IL-6), and Tumor Necrosis Factor-alpha (TNF-α)^55^, in the post-mortem brain tissue of individuals with schizophrenia^56^. Direct post-mortem studies further substantiate CNS involvement, reporting markedly elevated transcript levels for numerous immune-related markers and proinflammatory cytokines within the PFC^57^.

Microglia serve as multi-functional regulators of circuit stability beyond synaptic pruning^58,59^. Specifically, they are essential for proper myelination; altered microglial states can impair oligodendrocyte maturation and maintenance, directly contributing to the white matter microstructure abnormalities^60^ frequently observed in psychosis^61^. As the resident immune-related cells^62^, microglia regulate both excitatory^63^ and inhibitory inputs to pyramidal neurons^64^. To bridge the gap between genetic risk and cellular pathology, Experimental evidence from patient-derived cellular models, such as induced pluripotent stem cell (iPSC)-derived neurons, has definitively demonstrated that schizophrenia-linked C4 variants drive excessive synapse elimination^65^. in the psychosis spectrum, providing a cellular endotype that aligns with the dendritic spine deficits observed in post-mortem PFC tissue^66^.

## Linking Microglia to Cortical Circuitry Dysfunction

In a healthy PFC, balanced excitatory and inhibitory signaling ensures that relevant sensory information (the signal) is clearly processed^67,68^. Furthermore, recent transcriptomic studies suggest that the specific loss of these inputs disrupts the signal-to-noise ratio within cortical microcircuits, providing the biological basis for the ‘low precision’ signaling observed in computational models of psychosis^69^, where the brain can no longer distinguish between meaningful environmental cues and internal neural fluctuations, ultimately leading to the emergence of hallucinatory experiences^70^.

**Fig. 2:**
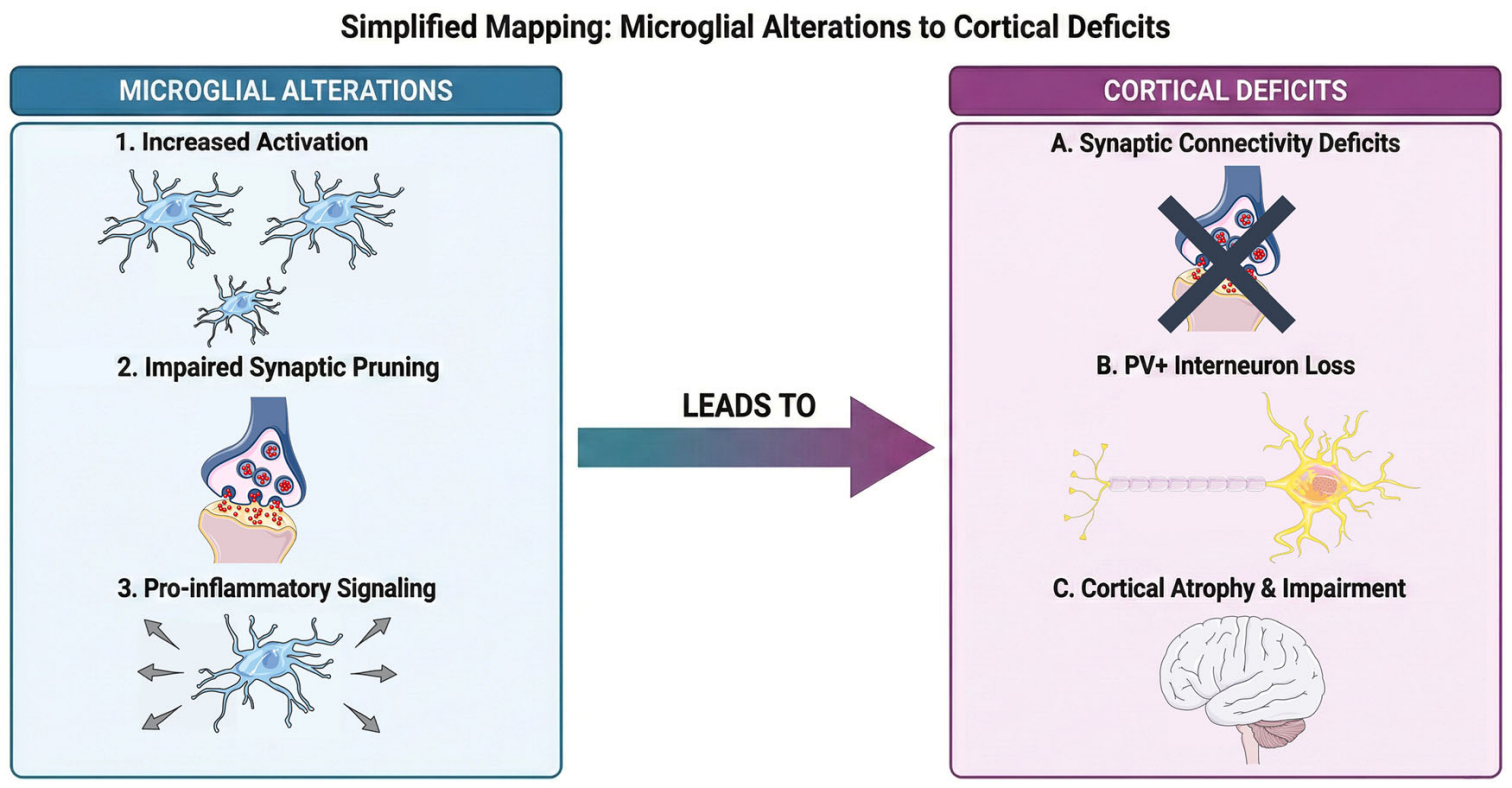
Simplified Mapping of Microglial Alterations to Cortical Deficits. his schematic illustrates the proposed causal chain where (1) increased microglial activation, (2) impaired synaptic pruning, and (3) pro-inflammatory signaling lead to downstream cortical impairments. These include (A) synaptic connectivity deficits via excessive C4-mediated elimination, (B) alterations or loss in PV+ interneuron signaling, and (C) macroscopic cortical atrophy and cognitive impairment.

Beyond excitatory inputs, disturbances are also noted in inhibitory neurons within the PFC, including deficits in transcript levels for the Gamma-aminobutyric acid-synthesizing enzyme glutamate decarboxylase (GAD67)^71,72^ and lower mRNA levels for the calcium-binding protein parvalbumin (PV)^73,74^. While microglial processes can displace perisomatic inhibitory terminals in animal models exposed to immune stimulants^75^, the density of PV basket cell terminals in the PFC of schizophrenia subjects was reportedly unchanged^76^, suggesting that generalized synaptic stripping of inhibitory inputs may not characterize the illness^75^ (Fig. 2).

The highly specific molecular finding that C4 confers risk by marking synapses for elimination^77^ implies that a generalized measure of microglial activation is insufficient; instead, research must focus on quantifying microglial endotypes specific to synaptic load regulation^78^. Heightened synaptic consumption is driven by specific microglial endotypes defined by markers like Complement Receptor 3 (CR3)^79^ and Ionized calcium-binding adaptor molecule 1 (Iba1)^80^. CR3 acts as the primary receptor that recognizes and binds to synapses, initiating phagocytosis^79^. Concurrently, Iba1—a protein involved in actin bundling^81^ serves as a marker of microglial motility and activation state, reflecting the cell’s capacity for structural remodeling^82^.

## Controversy and Heterogeneity in Neuroinflammation

Despite compelling genetic and peripheral evidence, post-mortem studies remain disparate, with some reports questioning a generalized role for neuroinflammation in schizophrenia at both the molecular and cellular levels^83,84^. “It is increasingly recognized that Translocater protein (TSPO) is a mitochondrial protein expressed not only in activated microglia but also in astrocytes and, crucially, is upregulated in neurons during periods of neuronal activity^85^. As demonstrated by Notter et al. (2021), this lack of cell-type specificity means that elevated TSPO signals in Positron Emission Tomography (PET) imaging may reflect changes in neuronal activity or homeostatic glial function rather than active neuroinflammation, necessitating the development of more selective microglial ligands^85^. (Table 2). Furthermore, in vivo imaging studies using TSPO ligands have yielded mixed results^86^. The current lack of cell-specific ligands complicates the interpretation of TSPO signals as purely microglial markers, necessitating a broader view of the neuroinflammatory milieu^87–89^.

**Table 2:** Analysis of Divergent Empirical Evidence and Methodological Heterogeneity in Neuro-Immune Psychosis Research.

Focus Area	Findings Supporting Neuro-Immune Role	Findings Questioning or Refining the Role
General Neuroinflammation	Post-mortem studies show markedly elevated proinflammatory cytokines (IL-1b, IL-6, TNF-α) in the PFC.	Some post-mortem reports question a generalized role for neuroinflammation at both molecular and cellular levels.
TSPO PET Imaging	Some in vivo imaging studies using TSPO ligands have shown elevated signals, suggesting glial activation.	Other TSPO studies have yielded mixed results. TSPO is not cell-specific
Inhibitory Synaptic Loss	Animal models exposed to immune stimulants show microglial processes displacing inhibitory terminals.	density of PV basket cell terminals in the PFC was unchanged, suggesting no generalized stripping of inhibitory inputs.
Clinical Efficacy of Minocycline	Some studies show “significant superiority” over placebo in improving positive, negative, and general symptoms.	Other trials found efficacy primarily concentrated in negative symptoms.
Other Anti-inflammatory Agents	Aspirin has shown a significant effect in some research contexts.	Celecoxib and n-3 Omega-3 polyunsaturated fatty acid (n-3 PUFA) have yielded inconsistent results and lacked significant effects in some trials.

To overcome the limitations of TSPO, research is shifting toward next-generation ligands^90^. While TSPO provides a high-sensitivity but low-specificity signal of glial activation^91^, newer targets like the Colony Stimulating Factor 1 Receptor (CSF1R) are being explored for their superior microglial specificity^92^. Furthermore, combining PET imaging with Magnetic Resonance Spectroscopy (MRS) to measure glutathione or glutamate levels allows for a more nuanced ‘neuro-immune signature’ that separates active metabolic signaling from static microglial density^93,94^. Conflicting findings in neuroinflammation research likely stem from methodological heterogeneity, including variations in TSPO ligand affinity (high vs. low affinity binders)^87,95^ and the inclusion of patients at different illness stages^96^.

## Neurodevelopmental and Environmental Interactions with Immune Liability

**Fig. 3:**
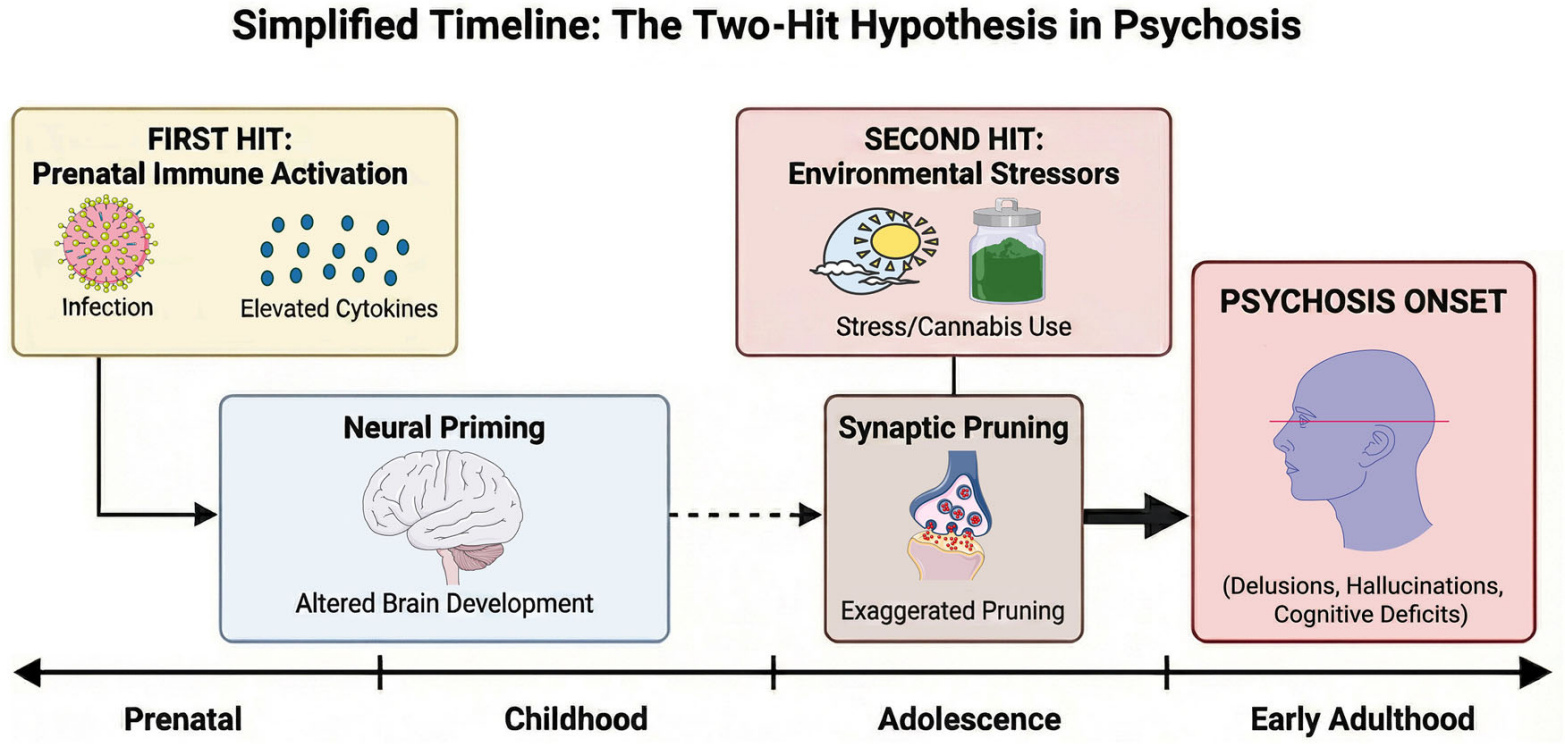
Simplified Timeline: The Two-Hit Hypothesis in Psychosis. A “First Hit” during the prenatal period (e.g., maternal infection/elevated cytokines) leads to neural priming and altered brain development. Subsequent “Second Hits” during adolescence, such as psychosocial stress or cannabis use, interact with this primed state. (3) This interaction drives exaggerated synaptic pruning, ultimately resulting in the clinical onset of psychosis in early adulthood.

The etiology of psychosis is fundamentally shaped by the timing of immune insults during critical neurodevelopmental windows. Longitudinal human studies and animal models of maternal immune activation (MIA) demonstrate that prenatal exposure to pathogens triggers a persistent pro-inflammatory state that alters fetal brain development^97,98^. These early-life events ‘prime’ the immune system, leading to long-term neural outcomes^99^ such as disrupted cortical migration and reduced synaptic density, which manifest during the late-adolescent peak of synaptic pruning^99^ (Fig. 3).

Conversely, elevated anti-inflammatory signaling in utero appears protective, correlating with a reduced risk of adult-onset psychosis^100^. The complexity of early-life immune priming is further illuminated by mouse models where pathological traits resulting from prenatal immune activation, such as reduced sociability and elevated cued fear, were observed to persist across multiple generations. This suggests that environmental insults do not merely affect the immediate offspring but can exert a persistent, transgenerational influence^101^. Such findings imply that the ‘immune liability’ for psychosis may be cumulative, where ancestral environmental stressors interact with current genetic vulnerability to shape the individual’s neurodevelopmental trajectory^101^ (Table 3).

**Table 3:** Cross-Species Synthesis of Neuro-Immune and Computational Phenotypes in Psychosis.

Finding Domain	Humans	other Models
Genetic Drivers	Polygenic risk scores (PRS) accounts for up to 80% heritability; focus on C4 variants and MHC complex.	C4-linked excessive synapse elimination validated in patient-derived iPSC models and transgenic rodents.
Environmental Insults	Epidemiological link between prenatal infection, adolescent stress, and adult psychosis.	MIA prove causality between prenatal infection and altered brain developmen in animal models.
Neuro-Immune Markers	Elevated IL-1β, IL-6, and TNF-α in post-mortem PFC tissue.	MIA offspring exhibit persistent pro-inflammatory states and diurnal-specific microglial abnormalities in preclinical models.
Clinical/Behavioral Manifestations	High-level positive symptoms: hallucinations and delusions.	Transgenerational Reduced sociability and elevated cued fear in mouse models

## Polygenic Risk, Chronic Stress, and Neuroanatomy

Genetic vulnerability interacts dynamically with diverse modifiable environmental factors, including post-infectious inflammation and chronic psychosocial stress^102^. There is a Suggestive but limited evidence pointing to gene-environment interactions between schizophrenia PRS and measures of chronic stress, such as hair cortisol^103^.

The joint influence of PRS and chronic stress—manifested as elevated cortisol—drives specific neuroanatomical remodeling^103^. High-PRS individuals exposed to chronic stress exhibit structural alterations, including total ventricle enlargement and impaired global white matter microstructure^103^. These structural alterations are most pronounced in regions critical for higher-order cognition, including the PFC and the hippocampus^104^ (Table 4). Increased mean diffusivity in global white matter suggests a breakdown in the structural integrity of the ‘brain’s wiring,‘^105^. The enlargement of the lateral ventricles serves as a macroscopic marker of this underlying gray^106^ and white matter loss^107^.

**Table 4:** Regional Neuro-Immune Pathology and its Computational Consequences in Psychosis.

Brain Region	Pathological Mechanism / Finding	Impact on Information Processing	Key Evidence / Sources
PFC	Microglial-mediated pruning of excitatory synapses; elevated pro-inflammatory cytokines (IL-1β, IL-6, TNF-α).	Reduces synaptic gain and sensory precision, leading to “maladaptive priors” and delusions.	Post-mortem transcript levels; C4-linked synapse elimination in iPSC models.
Hippocampus	Region-specific structural remodeling and neuroanatomical alterations driven by high polygenic risk and chronic stress.	affect higher-order cognition.	Macroscopic: ventricle enlargement Microscopic: impaired white matter structure
Cortical Microcircuits	Disrupted signal-to-noise ratio due to loss of specific excitatory inputs.	Inability to distinguish meaningful environmental cues from internal neural fluctuations.	Transcriptomic studies suggesting low-precision signaling.

## Translational Biomarkers in the At-Risk Mental State (ARMS)

The identification of reliable biomarkers is critical for early prediction and intervention, particularly in individuals presenting with ARMS, who exhibit attenuated psychotic symptoms or brief intermittent psychotic episodes^103^. ARMS offers a critical window for intervention defined by a distinct biomarker profile spanning multiple biological domains^108^. These markers can be categorized into peripheral cytokines^108,109^, metabolic signatures^109^, and neuroanatomical indicators that predict transition to full psychosis^110^ (Table 5). Specifically, low serum levels of 3-hydroxykynurenine (3-OHKY) have been associated with the greatest improvement in symptoms^111^. Intervention during an early period holds the greatest promise for altering the illness trajectory.

**Table 5:** Multimodal Biomarkers Predicting Psychosis Transition Risk.

Category	Specific Marker	Predictive / Clinical Significance
Peripheral Markers	Proinflammatory Cytokines (e.g., C-reactive protein/CRP > 3, IL-6, TNF-α)	Elevated levels indicate a “high-inflammatory biotype” an immunomodulation.
Metabolic Signatures	3-OHKY	Low serum levels are associated with significant improvement in symptoms.
Neuroanatomical Indicators	Lateral Ventricle Enlargement	Serves as a macroscopic marker for gray and white matter loss.
Neuroanatomical Indicators	Global White Matter Microstructure	Impaired integrity (increased mean diffusivity) indicates a breakdown in “brain wiring”.
Genetic / Imaging Integration	Magnetic resonance imaging (MRI) Pattern Recognition + PRS	Combined integration of MRI and PRS predicts high-risk individuals with 85.9% accuracy.
Digital Biomarkers	Environmental Predictability (Mobility patterns + social behaviour)	A decrease in environmental predictability can occur several days prior to clinical exacerbation.
Digital Biomarkers	Circadian / Sleep Abnormalities	Detectable via wearables, these drive circuit instability and signal functional instability.

## Translational Advances: Computational Modeling and Digital Biomarkers

Computational models provide a powerful framework for understanding positive psychotic symptoms, interpreting them not merely as subjective experiences but as consequences of fundamental errors in information processing^24^. Within the predictive coding framework, these symptoms, such as delusions and hallucinations, are modeled as a combination of maladaptive (or overly fixed) priors and reduced updating of these priors based on new sensory input^45^. This ability of computational models to link disparate explanatory levels—from neurobiological dysregulation, such as reduced synaptic precision, to high-level alterations in self-environment perception—is key to improving the mechanistic understanding of psychosis. Moreover, this modeling approach guides therapeutic research by generating hypotheses for new treatments derived from first principles^39,70^.

Sleep disturbances, detectable via wearable sensors^112^, are not merely symptoms but active drivers of circuit instability that alter microglial states in mice models^113^. Preclinical models of MIA demonstrate that adult offspring exhibit diurnal-specific microglial abnormalities^114^. While earlier research in digital phenotyping was characterized by methodological heterogeneity, recent large-scale longitudinal studies have established high predictive validity for relapse using these specific, passive behavioral metrics^115^. Operationally, ‘digital signatures’ of relapse often manifest as a decrease in environmental predictability—such as mobility patterns social behavior—occurring several days prior to clinical symptom exacerbation. This transition toward standardized sensor outputs and duration ensures that digital biomarkers are not only robust but also clinically valid across diverse global populations^116^. Moving toward clinical feasibility, future implementation must adopt standardized frameworks for data harmonisation^117^ and address the ‘digital divide’ by ensuring that tool design accounts for varying levels of cognitive load and digital literacy in patient populations^118^. This involves developing simplified, “user-friendly” interfaces^118^. Furthermore, implementation strategies must include digital literacy training as a core component of coordinated specialty care, ensuring that patients from diverse socioeconomic backgrounds can equitably access and benefit from remote monitoring technologies^118^. The complexity of psychosis pathology necessitates the integration of high-dimensional, multi-modal data streams for effective clinical prediction^119^. The reliance on standard clinical interviews alone proves insufficient for predicting individual patient response to antipsychotic drugs^119,120^. Therefore, the development of sophisticated predictive tools is essential for optimizing treatment plans early^121^. For instance, studies have demonstrated that the reliable early prediction of psychosis, particularly in high-risk individuals, can be enhanced by integrating neuroanatomical pattern recognition using MRI with genetic data, such as PRS with accuracy of 85.9% (sensitivity, 84.6%; specificity, 87.3%)^122^. This integration of genetics, imaging, and computational methods represents the necessary transition toward optimizing personalized treatment pathways^122^.

## Treatment Implications and Health-Systems Dimensions

This review synthesizes an interdisciplinary framework where neuro-immune dysregulation—specifically microglial-mediated synaptic pruning—serves as the biological catalyst for the computational deficits that define psychosis^48^. The transition from traditional nosology to a mechanism-driven framework requires resolving the ‘diagnostic silence’ regarding the causal chain between microglial activity and clinical symptoms^48^. By adopting the Research Domain Criteria (RDoC), researchers can move beyond broad diagnoses like ‘schizophrenia’ to focus on specific functional constructs, such as ‘cognitive systems’ or ‘negative valence systems.‘^123^. For example, instead of treating ‘thought disorder’ as a single symptom, an RDoC approach quantifies the underlying ‘reduced sensory precision’ via behavioral tasks^124,125^. This effectively breaks the diagnostic silence, allowing for treatments that target the biological driver rather than the clinical label^126,127^.

While current diagnostic categories rely on phenomenological traits, the integration of RDoC allows for the definition of phenotypic components based on physiological traits^126–128^. Specifically, we propose that the observed loss of basilar dendritic spines on deep layer 3 pyramidal neurons in the PFC serves as the structural bridge to cognitive impairment^129,130^. Accurate sleep data, obtainable through wearable sensors^112^, is particularly relevant because sleep disturbances can alter microglial states and trigger peripheral immune shifts^113^. By capturing real-time sleep-wake cycles, computational models can better predict symptom exacerbation and functional stability. Digital phenotyping offers a unique, real-time window into core features of psychosis, such as circadian and sleep abnormalities that actively drive circuit instability^131^. These digital data streams can be inverted to infer latent parameters like precision weighting^132^. For example, a marked decrease in environmental predictability may reflect a patient’s inability to accurately weigh sensory precision, acting as a measurable precursor to functional instability^41,133^. By capturing these rhythmic neuro-immune fluctuations, computational models can better predict symptom exacerbation^134,135^.

Despite the translational potential of these models, the field must resolve significant technical inconsistencies regarding neuroinflammation^136,137^. The reliance on TSPO imaging remains controversial because it lacks cell specificity, as evidenced by its expression in multiple CNS cell types^87–89^. Effective stratification requires moving beyond binary diagnoses toward a ‘high-inflammatory biotype’ model^21,138^. Clinical implementation would involve screening patients for a specific panel of peripheral markers—primarily CRP, IL-6, and TNF-alpha—where only those exceeding a predefined inflammatory threshold (e.g., CRP > 3 mg/L) are considered candidates for adjunctive immunomodulation^139,140^. This approach prevents the ‘dilution’ of treatment effects seen in broad clinical trials and ensures that agents like minocycline are reserved for individuals whose symptoms are biologically driven by active neuro-immune signaling^141^. Conversely, psychosocial interventions like Coordinated Specialty Care (CSC) provide the external “structure” and predictability that compensate for internal computational deficits in sensory precision^134^.

In conclusion, this integrated framework offers a path toward a truly personalized, biologically grounded psychiatry. While hurdles remain—including data heterogeneity and the need for global standardization—the proposed roadmap prioritizes molecular stratification, computational calibration, and digital intervention to alter the illness trajectory. Future research must prioritize the integration of longitudinal patient-derived cellular models with real-time digital metrics to validate these computational models in the “wet lab”. By overcoming these barriers, we can ensure that the transition to precision medicine is both equitable and accessible among various populations.

The neuro-immune hypothesis has spurred numerous clinical trials investigating immunomodulatory agents as adjunctive treatments for psychosis^142^. Minocycline, a non-selective microglial inhibitor^143^, has been extensively studied, yielding complex results that suggest efficacy is primarily concentrated on specific symptom domains, positive [Standardized Mean Difference: −0.15], negative [Standardized Mean Difference: −0.62]^144^. While traditional antipsychotics remain the gold standard for positive symptoms, they often fail to address negative symptoms and cognitive deficits^68^. In contrast, immunomodulators like minocycline and certain non-steroidal anti-inflammatory drugs show the greatest promise in improving cognitive^44^ and negative domains^145^, likely by stabilizing the synaptic pruning processes in the PFC^44,77^ (Table 6).

**Table 6:** Comparative Efficacy of Pharmacological and Psychosocial Interventions.

Intervention	Strategy Type	outcome
Minocycline	Pharmacological	Negative: *p* = 0.001; Positive: *p* = 0.13; Cognitive: mixed results (*p* = 0.47 to *p* = 0.52). Mixed results efficacy in negative symptoms
Traditional Antipsychotics	Pharmacological	Positive: Gold Standard; Negative/Cognitive: Often fail to address.
Aspirin	Pharmacological	Significant effect reported.
Celecoxib & n-3 PUFA	Pharmacological	No significant effect.
CSC	Psychosocial	Sensory/Functional: High effectiveness in improving predictability.
Assertive community treatment (ACT)	Psychosocial	Social/Functional: Effective for general stability. counteracts computational deficits in self-environment interaction.

Across multiple trials minocycline reveals significant heterogeneity, with some trials showing improvement in negative symptoms (*p* = 0.001) but no change in positive symptoms (*p* = 0.13), attention (*p* = 0.47), or memory (*p* = 0.52)^146^. and other studies showing “significant superiority” of minocycline over the placebo in improving positive, negative and general symptoms (*P* = 0.02–0.00001)^147^. Nonetheless, adjunctive use may still be considered for individuals with prominent negative symptoms^148^ or documented inflammation, highlighting the necessity for patient stratification^149^. Other anti-inflammatory approaches have also yielded inconsistent results, with aspirin showing signifianct effect but not Celecoxib and n-3 PUFA^150^.

Precision treatment in psychosis requires a clear distinction between molecular targeting and environmental restructuring^151,152^. From a health-systems perspective, the initial investment in multimodal biomarkers and digital phenotyping is offset by the substantial reduction in hospital readmission rates^153,154^. Early prediction and targeted intervention—such as applying immunomodulation only to ‘high-inflammatory’ biotypes—avoids the ‘trial-and-error’ approach that often leads to treatment resistance^155,156^. Long-term economic models suggest that preventing even a single psychotic relapse can save significant healthcare costs, making precision pathways a fiscally responsible alternative to traditional, non-stratified care^157^. For instance, therapeutic programs like CSC^158^ in early psychosis and ACT contain strong social elements^157^. Computational models propose that the observed efficacy of these interventions stems from the structure and predictability they introduce into the patient’s environment^134^. This environmental stability and structure are thought to directly counteract the perceived “low precision” afforded to sensory and environmental information that characterizes psychosis, thereby providing external cognitive resources that aid patients in regulating self-environment interactions and leading to symptom reduction^134^. Furthermore, systems-level challenges like poor adherence have been addressed through pharmaceutical engineering innovation, such as the development of weekly, prolonged-release oral capsules. This technology maintains consistent drug levels in the body, providing stability and ensuring patients reliably receive their medication, which is a fundamental requirement for successful clinical management^159^.

As artificial intelligence (AI) and computational methods become central to clinical decision-making and prediction in psychiatry, the major challenge of algorithmic bias must be proactively addressed^160,161^. Computational methods, rather than inherently eliminating human bias, are prone to reifying and magnifying existing health disparities if they are trained on non-representative datasets^162^. Many biomedical datasets suffer from the historical underrepresentation of certain patient groups, particularly ethnic and gender minorities^163^. Training algorithms on such skewed data risks fatal outcomes, misdiagnoses, and a lack of generalization, especially for marginalized individuals. This problem highlights that the pursuit of precision psychiatry is ethically inseparable from the pursuit of health equity^164^. Implementation should move beyond general open science to include ‘fairness-aware’ machine learning techniques that audit algorithms for disparate impact across ethnic and gender strata^165^. Furthermore, adopting the FAIR (Findable, Accessible, Interoperable, and Reusable) data principles ensures that the multi-modal datasets used for training are sufficiently diverse to permit generalization across global patient cohorts^166^. The future of precision psychiatry hinges on reconciling the temporal dynamics of neuro-immune activity with the real-time fluctuations of clinical symptoms^167^. Rather than treating psychosis as a static biological destiny, we must view it as a fluid state where circadian disruptions and sleep architecture abnormalities act as primary drivers of circuit instability^168,169^. These rhythmic fluctuations, detectable via non-invasive wearable sensors, provide a unique window into the “state-dependent” nature of neuroinflammation that traditional cross-sectional interviews inevitably miss^132^. However, as we pivot toward these high-dimensional predictive models, we encounter a second frontier: the ethical imperative of algorithmic fairness^170^. To prevent reifying historical health disparities, the implementation of psychiatric AI must strictly adhere to Consolidated Standards of Reporting Trials–Artificial Intelligence (CONSORT-AI)^171^ and Standard Protocol Items: Recommendations for Interventional Trials-Artificial Intelligence (SPIRIT-AI) reporting standards, ensuring that “precision” does not come at the cost of equity for marginalized populations^172^.

## Data Availability

The original contributions presented in the study are included in the article/supplementary material; further inquiries can be directed to the corresponding authors.
